# Discovery of highly potent and novel LSD1 inhibitors for the treatment of acute myeloid leukemia: structure-based virtual screening, molecular dynamics simulation, and biological evaluation

**DOI:** 10.3389/fphar.2025.1510319

**Published:** 2025-02-27

**Authors:** Ye Hong, Yuting Wang, Ziyi Hao, Xingxia Zhang, Yejun Si, Guoqiang Lin, Shurong Zhang, Miao-Miao Niu, Xiaotian Yang, Yanming Zhang

**Affiliations:** ^1^ Department of Hematology, The Affiliated Huai’an Hospital of Xuzhou Medical University, Huai’an, Jiangsu, China; ^2^ Department of Hematology, Binhai Couty People’s Hospital, Yancheng, Jiangsu, China; ^3^ Department of Pharmaceutical Analysis, China Pharmaceutical University, Nanjing, Jiangsu, China

**Keywords:** acute myeloid leukemia, LSD1 inhibitors, structure-based virtual screening, molecular dynamics simulation, biological evaluation

## Abstract

Acute myeloid leukemia (AML) is a highly aggressive hematological malignancy with a significant unmet clinical need for new therapeutic agents. Lysine-specific demethylase 1 (LSD1), a key regulator of leukemia stem cell self-renewal, has emerged as a promising epigenetic target for AML treatment. Herein, we employed an innovative multi-step integrated screening protocol, encompassing pharmacophore modeling, docking screening, molecular dynamics simulation, and biological evaluation, to identify novel LSD1 inhibitors. This comprehensive approach led to the discovery of six potent LSD1 inhibitors (we named these inhibitors LTMs 1–6), with LTM-1 exhibiting the most pronounced inhibitory effects on LSD1 (IC_50_ = 2.11 ± 0.14 nM) and the highest selectivity for LSD1 over LSD2 (>2370-fold). Notably, LTM-1 demonstrated outstanding antitumor activity both *in vitro* and *in vivo*. *In vitro*, LTM-1 showed potent anti-proliferative effects against LSD1-addicted MV-4-11 leukemia cells (IC_50_ = 0.16 ± 0.01 μM). *In vivo*, LTM-1 treatment significantly reduced tumor growth in MV-4-11 xenografted mice. Moreover, LTM-1 did not induce significant changes in liver and kidney function indices, suggesting a favorable safety profile. These results indicate that LTM-1 is a highly promising preclinical candidate for AML treatment, offering a new strategy for the development of more effective and selective LSD1 inhibitors.

## 1 Introduction

Acute myeloid leukemia (AML) accounts for about 80% of all adult acute leukemia patients and is the most common type of acute leukemia in adults (Dong et al., 2020). AML is a heterogeneous hematological malignancy characterized by abnormal clonal expansion of hematopoietic progenitor cells with differentiation defects (Miles et al., 2020; Rubnitz et al., 2010; Prada-Arismendy et al., 2017). With a mortality rate of up to 60%, AML is one of the deadliest leukemias (Jani et al., 2023). In the past 30 years, despite significant progress in traditional cytotoxic chemotherapy, targeted therapy, and immunotherapy for AML, the number of newly diagnosed leukemia patients worldwide has increased by 46% annually due to aging and the widespread use of chemotherapy leading to secondary leukemia (Wei et al., 2023; Martínez-Cuadrón et al., 2022). Furthermore, the 5-year overall survival rate of patients with AML is less than 50% (Shallis et al., 2019; Tang et al., 2024). With the rapid development of molecular cancer biology, more precise and personalized targeted therapy strategies have shown great potential (Newell and Cook, 2021). However, considerable challenges persist in overcoming treatment resistance and improving the efficacy of AML therapies. There is an urgent and unmet clinical need for the development of novel therapeutic agents that can significantly enhance the prognosis and survival of AML patients.

As the first discovered histone demethylase in 2004, LSD1 is a member of the flavin adenine dinucleotide (FAD)-dependent amine oxidase demethylase family, and another isoform LSD2 was subsequently discovered in 2009 (Shi et al., 2004; Ciccone et al., 2009). LSD1 catalyzes the demethylation of histone 3 lysine 4 methyl 1/2 (H3K4me1/2) and histone 3 lysine 9 methyl 1/2 (H3K9me1/2) (Metzger et al., 2005). Additionally, LSD1 has been found to catalyze the demethylation of some non-histone lysines, including p53, STAT3, E2F transcription factor 1, and DNA methyltransferase 1 (DNMT1), thereby regulating their downstream cellular functions (Xie et al., 2011; Huang et al., 2007; Wang et al., 2008). However, abnormal overexpression of LSD1 has been found in various hematological diseases, including AML, acute lymphocytic leukemia (ALL), myeloproliferative tumors, and chronic myelomonocytic leukemia. Particularly in AML, LSD1 is overexpressed in approximately 60% of cases (Niebel et al., 2014). Moreover, Harri et al. have elucidated that LSD1 is a critical regulator for the self-renewal of leukemia stem cells using the human MLL-AF9 leukemia model (Harris et al., 2012). Furthermore, previous studies have shown that the compound effectively inhibiting LSD1 exhibits synergistic activity with anti-leukemic drugs (Binda et al., 2010). In addition, LSD1 inhibition can enhance the sensitivity of AML cells to all-trans retinoic acid (ATRA) treatment, which was first used to treat AML by inducing cell differentiation (Lokken and Zeleznik-Le, 2012; Schenk et al., 2012). These findings collectively underscore the promise of LSD1 as a highly promising epigenetic target for the treatment of AML (Zhang et al., 2021), providing a compelling biological basis for our investigation.

**FIGURE 1:**
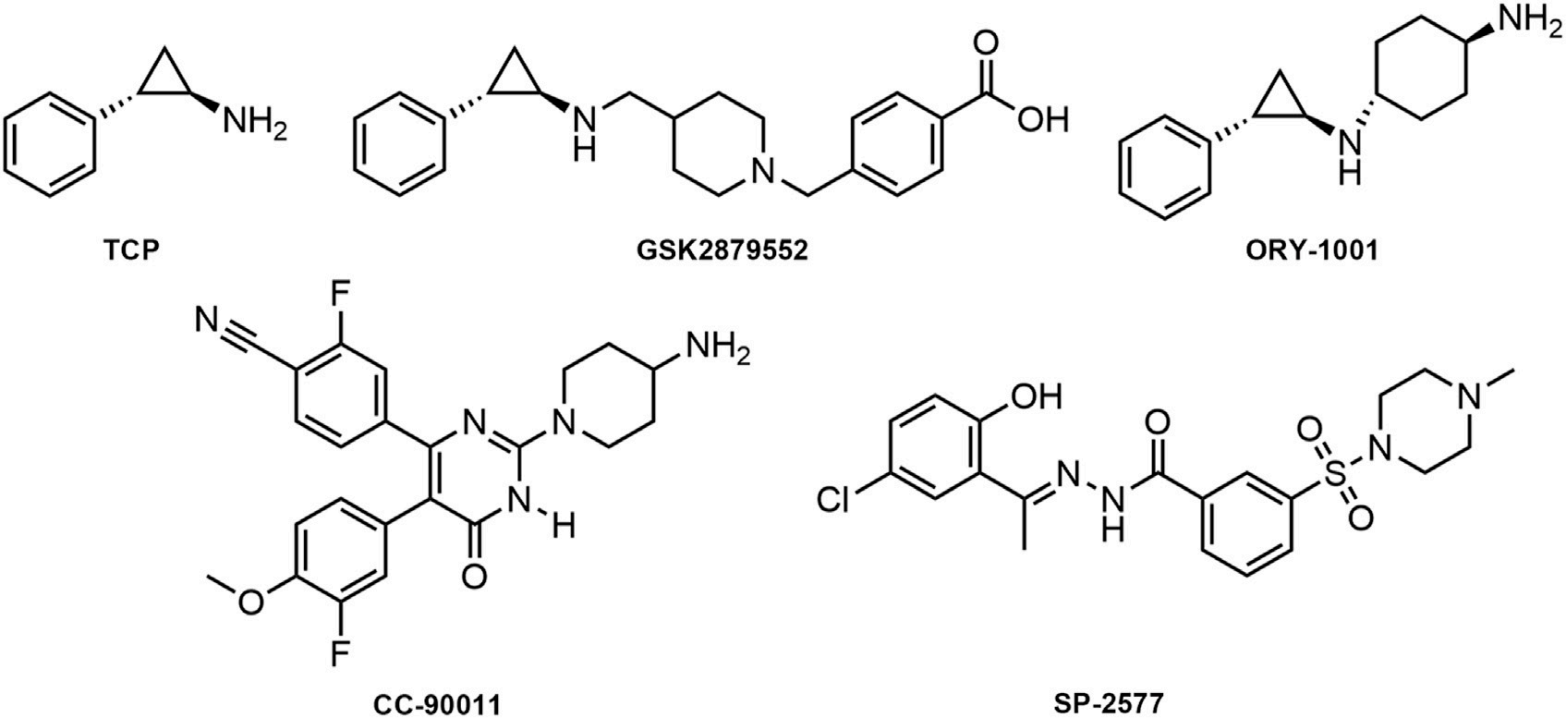
Structures of published LSD1 inhibitors.

Given the central role of LSD1 in AML pathogenesis and its association with poor prognosis, targeting LSD1 presents a unique opportunity to develop a new class of therapeutics that could potentially overcome the limitations of current treatments. By inhibiting LSD1, we may be able to disrupt the self-renewal capacity of leukemia stem cells, a key factor in disease relapse, and also modulate the expression of critical genes involved in leukemia progression. This could lead to more effective and durable responses in patients, improving both survival rates and quality of life. Therefore, the motivation for our study stems from the need to develop more effective and selective LSD1 inhibitors. Tranylcypromine (TCP) has been identified as an irreversible and non-selective LSD1 inhibitor by forming a covalent TCP-FAD adduct (Figure 1) (Lee et al., 2006; Fioravanti et al., 2020). Based on the importance of the TCP scaffold in inhibiting LSD1 activity and the clinical demand for developing more effective and selective LSD1 inhibitors, various TCP derivatives have been synthesized (Ganesan et al., 2017; Schulz-Fincke et al., 2018; Koda et al., 2022). Currently, irreversible LSD1 inhibitors based on the TCP scaffold, including TCP, GSK2879552, and ORY-1001 are undergoing clinical trials for AML (Zheng et al., 2016; Huang et al., 2017; Somervaille et al., 2016). In addition, reversible LSD1 inhibitors such as CC-90011 and SP-2577 have also been evaluated in clinical trials (Kanouni et al., 2020; Kurmasheva et al., 2021). However, Sacilotto et al. conducted a comprehensive evaluation of the *in vitro* inhibitory potential of LSD1 inhibitors at clinical stages and found that SP-2577 had minimal effects on AML markers and did not exhibit satisfactory targeting of LSD1, thereby raising doubts about the efficacy of reversible LSD1 inhibitors in the treatment of AML (Sacilotto et al., 2021; Baby et al., 2023). In summary, there is still an urgent need for the development of highly selective LSD1 inhibitors inhibitors that can potently inhibit cancer cell proliferation without side effects for AML treatment.

The novelty of our approach lies in the application of a multi-step integrated screening protocol to identify novel LSD1 inhibitors. At present, structure-based virtual screening is widely utilized for identifying lead compounds and optimizing drug candidates, attributed to its high efficiency and cost-effectiveness (Aziz et al., 2022; Ballante et al., 2021). Pharmacophore-based screening, utilizing key pharmacophore elements, rapidly screens large databases to identify potential active molecules (Lanka et al., 2023). Molecular docking-based screening analysis evaluates the binding modes and affinities of target proteins with ligands for identification of candidate drugs (Raval and Ganatra, 2022). In prior studies conducted by our group, we successfully employed an integrated screening strategy combining pharmacophore modeling and molecular docking to discover effective inhibitors, including dual KRASG12D single targeted inhibitors and tubulin/NRP1 dual targeted inhibitors (Wang et al., 2022; Zheng et al., 2023). In this study, a multi-step integrated screening protocol encompassing pharmacophore modeling, docking screening, molecular dynamics simulation, and biological evaluation led to the identification of six novel LSD1 inhibitors for the treatment of myeloid leukemia (we named these inhibitors LTMs 1–6). Notably, LTM-1 and LTM-3 exhibited strong *in vitro* enzymatic inhibitory activity. In particular, LTM-1demonstrated outstanding antitumor activity both *in vitro* and *in vivo*. Overall, these results indicate that LTM-1 may be a promising preclinical candidate for further investigation.

## 2 Materials and methods

### 2.1 General

The compounds LTMs 1–6, identified in our study, and the positive control GSK2879552 were purchased from WuXi AppTec (Shanghai, China). These were utilized in both *in vitro* and *in vivo* assays. Iscove’s Modified Dulbecco’s Medium (IMDM), Roswell Park Memorial Institute (RPMI)-1640 medium, fetal bovine serum (FBS) and penicillin/streptomycin were purchased from Gibco (Grand Island, New Nork, United States). Recombinant human LSD1 (172–852 aa) and full-length LSD2 were obtained from Sino Biological Inc. and ActiveMotif.

### 2.2 Cell lines and culture conditions

The myeloid leukemia cell lines MV-4-11, MOLT-4, MOLM-16, HL-60, and the human B lymphocyte cell line RPMI-226 were purchased from the American Type Culture Collection (ATCC, Manassas, VA, United States). The myeloid leukemia cell line HAL-01 was obtained from the Deutsche Sammlung von Mikroorganismen und Zellkulturen GmbH (DSMZ, Braunschweig, Germany). All cells were cultured in IMDM or RPMI -1640 medium supplemented with 10% FBS, 100 U/mL penicillin, and 100 μg/mL streptomycin. The cells were maintained in a humidified incubator with an atmosphere of 5% CO_2_ at 37°C.

### 2.3 Establishment of a structure-based pharmacophore model for LSD1

The X-ray crystal structure of LSD1 complexed with the inhibitor CC-90011 (PDB ID: 6W4K) was obtained from the Protein Data Bank (PDB) and loaded into the molecular operating environment (MOE, Chemical Computing Group Inc, Montreal, Quebec, Canada). 6W4K was selected due to its high-resolution structure and relevance in previous studies related to LSD1 inhibitors (Kanouni et al., 2020). Within MOE, the QuickPrep tool was employed to execute a series of preparatory steps essential for refining the crystal structure. These steps included the elimination of unbound water molecules, the addition of polar hydrogen atoms, the computation of partial charges, and energy minimization. Subsequently, the Ligand Interaction tool in MOE was engaged to dissect the key key interactions between the LSD1 protein and its bound ligand. Finally, leveraging the detailed understanding of the LSD1 crystal structure, pharmacophore features were meticulously constructed using Pharmacophore Query Editor. These features encapsulate the critical interaction points necessary for the binding of inhibitors to LSD1, including hydrogen-bond donors (Don), hydrophobic features (Hyd), and aromatic centers (Aro).

### 2.4 Virtual screening

As described in the previously reported methods, virtual screening was performed using the MOE software (Liang et al., 2023). Initially, a database of 121,000 two-dimensional compounds assembled through combinatorial chemistry was converted into three-dimensional structures using an energy minimization algorithm. Subsequently, the prepared LSD1 pharmacophore features were used as a template for preliminary pharmacophore-based screening. Finally, the docking score of −8.53 kcal/mol, obtained from the LSD1 inhibitor GSK2879552, which is in the clinical trial phase, was used as a reference value for docking-based screening. The MOE docking scoring function was employed to evaluate the binding free energy of the ligand with the target protein. Compounds with scores lower than the set reasonable reference value were selected to obtain potential LSD1-targeting candidate inhibitors.

### 2.5 Molecular dynamics simulation

The crystal structure of LSD1 (PDB ID: 6W4K) was retrieved from the PDB. The crystal complex structures of the ligand LTM-1 with the protein LSD1 were constructed in the MOE and used as the initial coordinates for MD simulations. The ligand LTM-1 and the protein LSD1 were individually subjected to MD simulations using GROMACS (version 2021.5). LSD1 was topologically modeled under periodic boundary conditions, employing the AMBER99SB-ILDN force field for accurate representation. The Acpype Server (www.bio2byte.be) was engaged to generate the topology file of LSD1, which was then integrated with the ligand file to create a comprehensive complex system. Subsequently, the ligand file was added to the protein file to form a complex system. This complex system was immersed in a 1.0 nm cubic simulation box, employing the SPC water model to ensure solvation and equilibrium. To ensure system neutrality, Na^+^ and Cl^−^ were introduced. The solvated system underwent energy minimization, applying the steepest descent algorithm for 50,000 steps. A 100 ps NVT simulation was then performed on the system using the V-rescale thermostat to maintain the system temperature at 300 K. This was followed by a 100 ps NPT simulation, where the Parrinello-Rahman barostat was used to maintain a pressure of 1 bar. Finally, a 500 ns molecular dynamics simulation is conducted for each system, with trajectory data recorded at intervals of 0.1 ns. Data processing is performed using GraphPad Prism 10 software (GraphPad Software Inc., San Diego, CA), and stability is analyzed in conjunction with the root mean square deviation (RMSD), root mean square fluctuation (RMSF), binding-induced conformational changes and protein’s radius of gyration (Rg).

### 2.6 Binding free energy calculation using MM/PBSA method

The Molecular Mechanics/Generalized Born Surface Area (MM/GBSA) method was exploited through the GMX - PBSA tool for calculating the free binding energy of the LSD1 - LTM - 1 complex (Al-Khafaji and Tok, 2020). This computational tool included the PCM and SGB models for solvation energy calculations, along with consideration of van der Waals and electrostatic interaction energies to evaluate the overall energy within the complex. Finally, from the calculation results, we obtained the total binding Gibbs free energy (ΔGtotal). ΔGtotal comprises Van der Waals, Electrostatic, Polar solvation, Non - polar solvation, Net gas phase, and Net solvation. Van der Waals is the contribution of attractive or repulsive forces between molecules or within a single molecule. Electrostatic is the contribution of electrostatic interactions between charged particles within molecules. Polar solvation is the polar contribution to solvation free energy. Non - polar solvation is the non - polar contribution to solvation free energy. Net gas phase is the sum of internal energies of the isolated molecules in vacuum. Net solvation is the energy change when molecules interact with a solvent environment.

### 2.7 Enzyme inhibition assay

The inhibitory effects of the tested compounds on LSD1 and LSD2 enzymes were methodically assessed using the Lance Ultra LSD1 Histone H3-Lysine 4 Demethylase Assay Kit from PerkinElmer, adhering to previously published methodologies (Li et al., 2022). In brief, the reaction was initiated by adding 4 μL of enzyme solution, with concentrations specified at 2 nM for LSD1 or 172 nM for LSD2, 4 μL of substrate solution [2.5 μM Bio-H3K4me2 (124 amino acids)], and 2 μL of tested compound to a Tris buffer (50 mM Tris-HCl, 50 mM NaCl, 0.01% Tween 20, 1 mM DTT, 10 μM FAD, 10% glycerol, pH 9.0) at room temperature and incubated for 1 h. Following the incubation, 5 μL of a detection mixture, comprising an Eu-labeled H3K4me0 antibody and ULight Streptavidin, was added to the reaction. The fluorescence intensity was then measured employing TR-FRET mode with excitation and emission wavelengths set at 320 nm and 665 nm, respectively, utilizing the Envision (PerkinElmer) system. Each experiment was performed in triplicate.

### 2.8 Kinase selectivity assays

The kinase selectivity analysis of LTM-1 was performed utilizing the SelectScreen Kinase Profiling Service offered by Thermo Fisher Scientific. In a dose-response assay featuring a ten-point dilution series from 0.25 μM to 128 μM, the inhibitory effects of LTM-1 on substrate phosphorylation reactions catalyzed by a panel of kinases were assessed. Consequently, the half-maximal inhibitory concentrations (IC_50_ values) for each kinase were determined.

### 2.9 Cell proliferation assay

The *in vitro* anti-proliferative activity of the compounds was determined using the CellTiter 96^®^ AQueous NonRadioactive Cell Proliferation Assay Kit (Promega, Madison, United States) as previously described (Ji et al., 2017). In brief, 5–10 × 10^3^ cells and 180 μL of medium were first seeded into each well of a 96-well plate. Subsequently, the cells were exposed to either 0.2% Dimethyl sulfoxide (DMSO, Aladdin Reagent, Shanghai, China) or a serial dilution of the compounds, prepared from a 10 mM stock solution in DMSO, with the final concentration of DMSO being 0.2% for 72 h. 20 μL of MTS reagent was added to each well, and the culture was incubated in the dark at 37°C for 4 h. Finally, the optical density (OD) was then recorded at 490 nm and 690 nm using a microplate reader from Molecular Devices. The proliferation inhibition rate was calculated according to the following formula: inhibition ratio = (OD of DMSO treated wells - OD of compound treated wells)/(OD of DMSO treated wells - OD of blank wells) × 100%. The IC_50_ values of the compounds inhibiting cell proliferation were calculated using GraphPad Prism 10 (GraphPad Software Inc., San Diego, CA).

### 2.10 *In vivo* experiments

Given the potent inhibitory effect of LTM-1 on MV-4-11 cell proliferation *in vitro*, a previously reported xenograft animal model was established to measure the anti-tumor growth and development effects of LTM-1 (Zhou et al., 2022). The animal experimental design of this study was reviewed and confirmed by the Ethics Committee of China Pharmaceutical University. The experimental procedures complied with the ARRIVE guidelines (https://arriveguidelines.org/). Female BALB/c nude mice aged 4–6 weeks were purchased from Changzhou Cave Animal Co., Ltd. (Changzhou, China). Mice were housed in a specific pathogen-free (SPF) environment, where the temperature was precisely regulated to 25°C ± 2°C, the relative humidity was kept at 50% ± 5%, and a strict 12-h light-dark cycle was maintained. A 200 μL suspension of MV-4-11 cells (1 × 10^6^ cells) was injected subcutaneously into the mice. When the tumor volume was observed to reach 80–100 mm^3^, the mice were randomly divided into two groups: the vehicle group and the LTM-1 treatment group (10 mg/kg). The mice were administered drugs via intraperitoneal injection daily, with a total treatment period of 15 days. Mouse weight and tumor volume were measured every 3 days. The tumor volume was calculated using the formula (c × c × d)/2 (where c represents the smallest diameter, and d represents the largest diameter). When the animals could no longer access food and/or the average tumor diameter exceeded 15 mm, the mice were anesthetized with 2.5% isoflurane and euthanized by cervical dislocation to minimize suffering. In addition, the liver and kidney index levels were measured using an automatic biochemical analyzer to evaluate the toxic effects of LTM-1 on mice.

### 2.11 Statistical analysis

The entire statistical analysis was performed with GraphPad Prism 10. The t-tests were employed to evaluate whether there was a significant difference between the two sets of data. P values less than 0.05 were considered as significant. The data are presented as the mean ± standard deviation (SD), n = 3.

## 3 Results

### 3.1 Establishment of pharmacophore model

**FIGURE 2:**
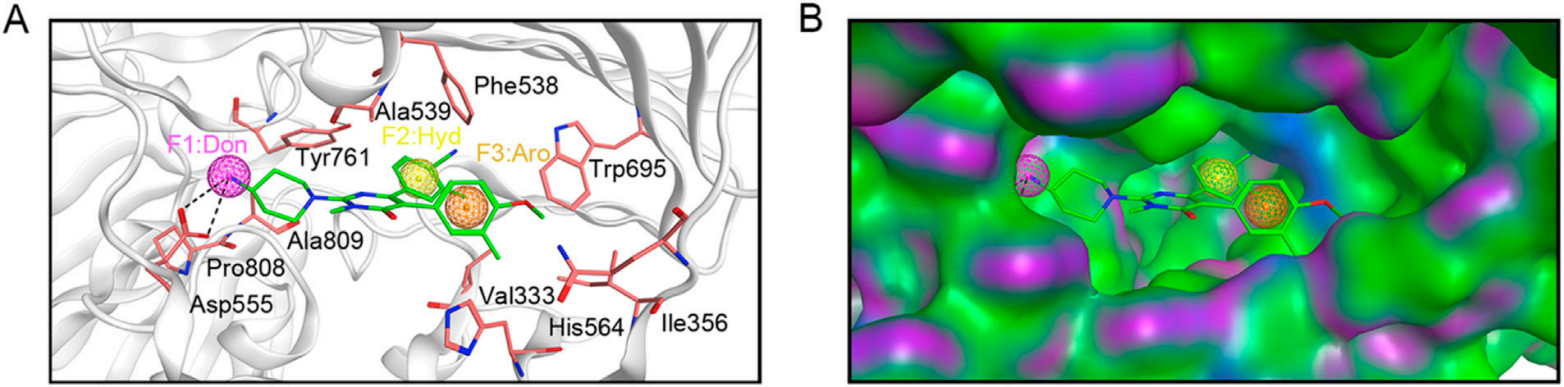
Pharmacophore model based on LSD1. **(A)** Details of pharmacophore model features. Hydrogen-bond donor (F1) is represented by magenta sphere, hydrophobic feature (F2) is depicted by yellow sphere, and aromatic center (F3) is illustrated by brown sphere. Each critical amino acid is represented by a pink stick model and annotated with a three-letter amino acid code. Hydrogen bonds are depicted with black dashed lines. **(B)** Spatial constraints of the pharmacophore model within the LSD1 active site.

Based on the crystal structure of LSD1 (PDB ID: 6W4K), a pharmacophore model was constructed using MOE to provide clues for identifying novel LSD1 inhibitors. Firstly, the LSD1 protein was preprocessed by removing water molecules, adding polar hydrogens, and calculating partial charges. Subsequently, the binding details of the LSD1 protein and its ligand were analyzed using the ligand interaction tool of MOE to yield key information for constructing the pharmacophore model. As depicted in Figure 2A, the ligand formed two hydrogen bonds with Asp555. Moreover, the ligand engaged in hydrophobic interactions with key amino acid residues Val333, Ile356, Phe538, Ala539, Tyr761, and Ala809 of LSD1. Based on the structure-activity relationship analysis of the LSD1 protein-ligand complex, three representative pharmacophore features were constructed using the pharmacophore query editor: a hydrogen-bond donor feature (F1) corresponding to the Asp555 residue; a hydrophobic interaction feature (F2) corresponding to the Phe538, Ala539, and Tyr761 residues; and an aromatic feature (F3) corresponding to the Trp695 residue. Additionally, the spatial constraints of the pharmacophore model within the LSD1 active site demonstrated that the established pharmacophore features were properly embedded into the depressions of the LSD1 binding pocket (Figure 2B). The constructed pharmacophore model offered critical chemical features for the subsequent virtual screening of novel LSD1 inhibitors.

### 3.2 Virtual screening

**FIGURE 3:**
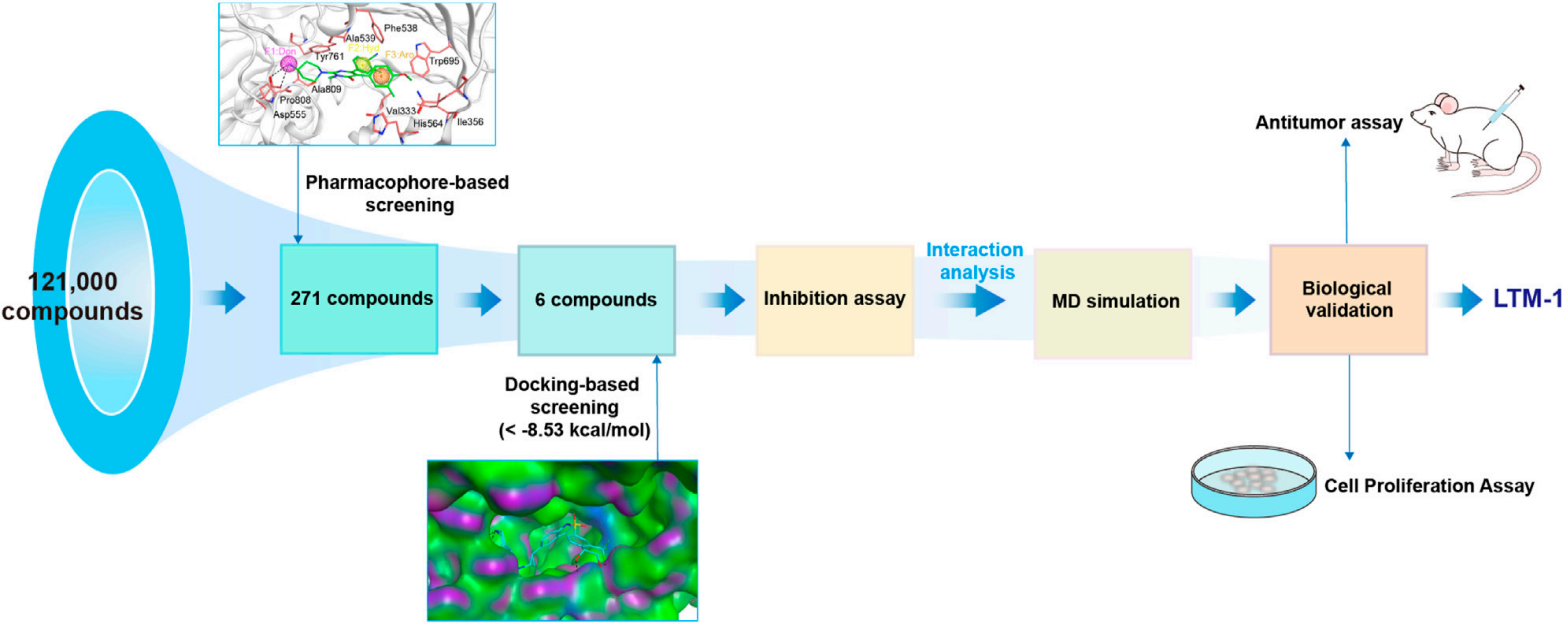
A flowchart of the identification of LSD1 inhibitors via an integrated strategy of *in silico* screening and biological evaluation.

**FIGURE 4:**
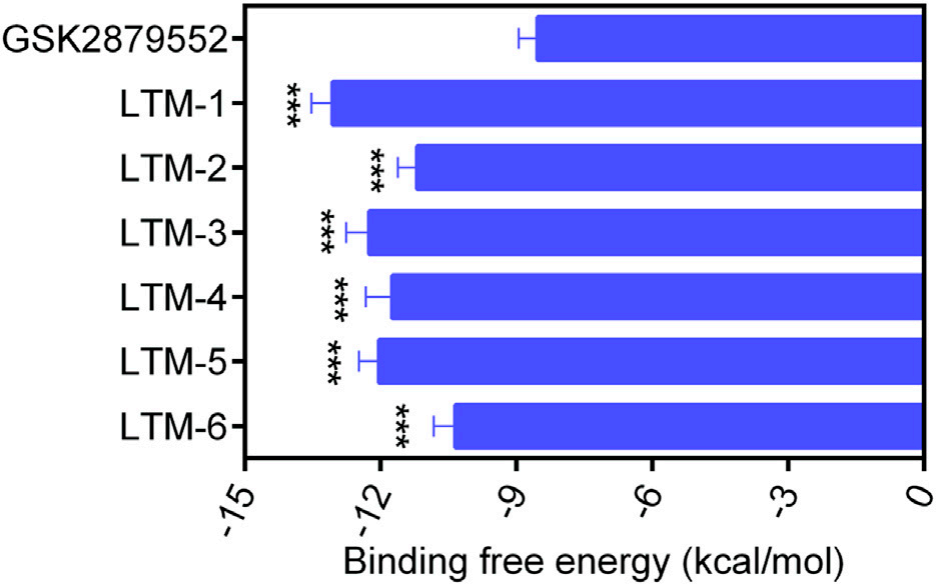
The docking scores of LTMs 1–6 and GSK2879552. ^a^Binding free energy between the compounds and the targets (lower binding free energies show stronger binding affinities). The data are presented as the mean ± SD, n = 3. ***p < 0.001, as compared to GSK2879552.

**FIGURE 5:**
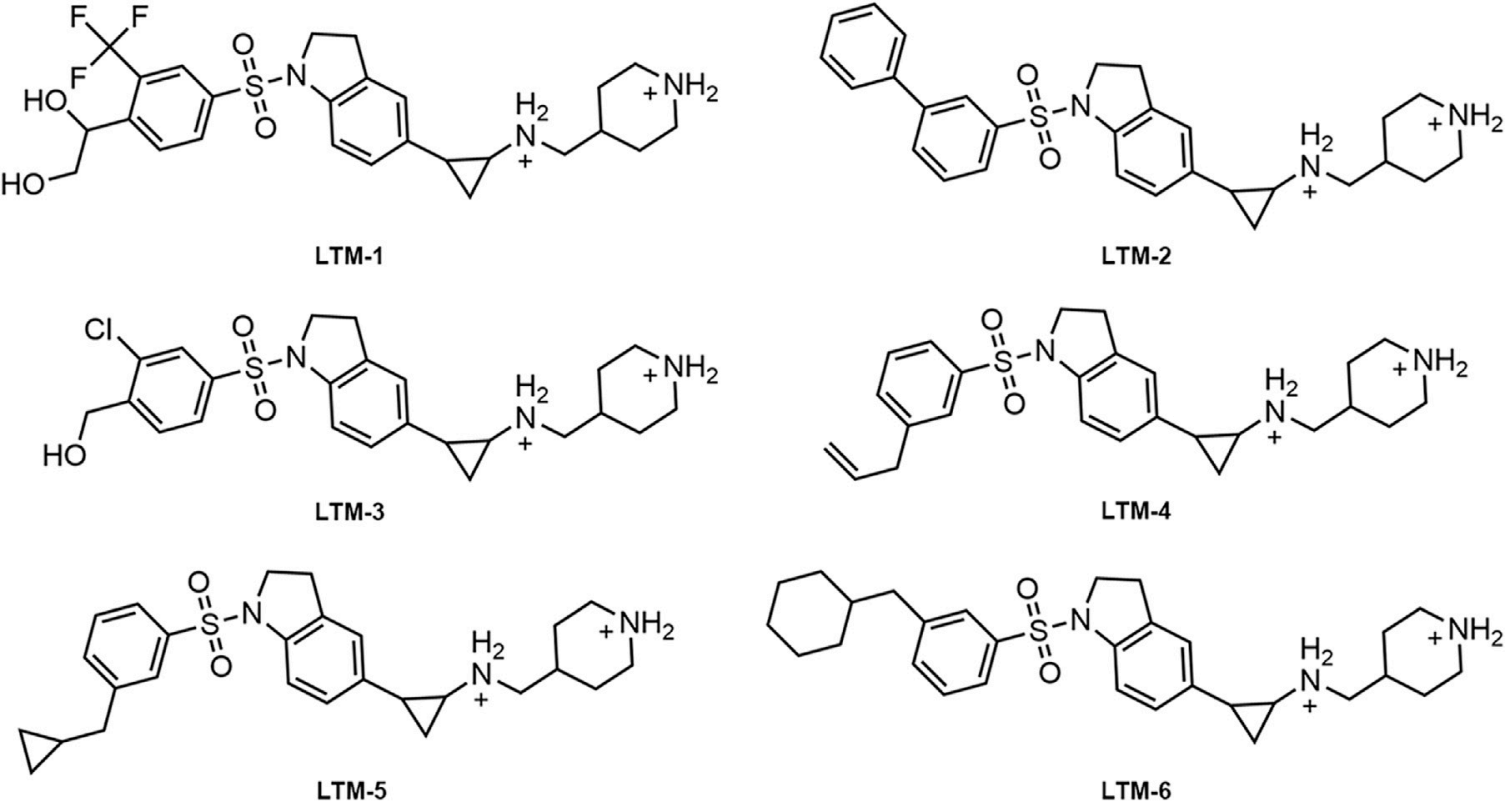
The chemical structures of LTMs 1–6.

As illustrated in Figure 3, this study employed a comprehensive screening strategy that integrated pharmacophore-based screening, molecular docking-based screening, molecular dynamics (MD) simulation, and biological evaluation to identify novel and potent LSD1 inhibitors. Initially, an energy optimization algorithm was applied to convert 121,000 compounds from a combinatorial chemistry-derived database from two-dimensional (2D) to three-dimensional (3D) structures. Subsequently, the LSD1 pharmacophore model was utilized as a probe for pharmacophore-based docking. The 207 compounds obtained through preliminary screening were docked to the LSD1 binding site for further docking-based screening and the binding free energy of each compound was calculated. The lower the binding free energy, the stronger the binding affinity between the compound and the target protein. Based on the docking score of the positive control GSK2879552 (−8.53 kcal/mol), which served as the threshold, the top six compounds (LTMs 1–6) were ultimately identified (Figure 4). The structures of these compounds are displayed in Figure 5, and according to SciFinder and Reaxys databases, LTMs 1–6 have not yet been reported internationally. Subsequently, these hit compounds were prepared for subsequent *in vitro* enzyme inhibition assays.

### 3.3 *In vitro* LSD1 inhibitory activity

The enzyme inhibitory activity of the six candidate inhibitors obtained was further determined. The LSD1 inhibitor GSK2879552, which has entered the clinical trial stage, was used as a positive control. As shown in Table 1, LTMs 1–6 exhibited nanomolar inhibition of LSD1 (IC_50_ = 2.11–16.54 nM), and LTMs 1–6 had stronger enzyme inhibition of LSD1 than GSK2879552 (IC_50_ = 24.53 ± 2.26 nM). Particularly, the enzyme inhibitory activities of LTM-1 (IC_50_ = 2.11 ± 0.14 nM) and LTM-3 (IC_50_ = 4.57 ± 0.32 nM) were the most prominent. Moreover, the enzyme inhibitory capacities of LTMs 1–6 against LSD2 were evaluated to determine the selectivity of their inhibition. The selectivity of LTMs 1–6 for LSD1 over LSD2 was superior to that of GSK2879552, with LTM-1 (>2370-fold) and LTM-3 (668-fold) showing the most significant selectivity. In addition, the excellent enzyme inhibitory activity and selectivity of LTM-1 and LTM-3 are consistent with the lowest docking scores of LTM-1and LTM-3 with LSD1 mentioned above. Additionally, the compound with the most potent inhibitory activity and selectivity against LSD1, LTM-1, underwent comprehensive kinase panel analysis to assess its broad-spectrum inhibition potential against 60 different kinases. The data presented in Supplementary Table S1 demonstrate that the IC50 values of LTM-1 against each of the 60 evaluated kinases were all greater than 10 μM. Overall, these results indicate the significant selectivity of LTM-1 for LSD1, reducing the risk of off-target effects and associated toxicity.

**TABLE 1:** The inhibitory activities of LTMs 1–6 against LSD1 and LSD2.

Compounds	LSD1 (IC_50_, nM)	LSD2 (IC_50_, nM)	LSD2/LSD1 selectivity (folds)
LTM-1	2.11 ± 0.14	>5,000	>2,370
LTM-2	10.23 ± 2.71	2,196.02 ± 146.25	215
LTM-3	4.57 ± 0.32	3,054.75 ± 177.86	668
LTM-4	8.92 ± 0.57	861.49 ± 26.73	97
LTM-5	7.31 ± 0.46	673.67 ± 21.48	92
LTM-6	16.54 ± 2.39	>5,000	>302
GSK2879552	24.53 ± 2.26	103.56 ± 5.75	4

Data represent mean values ± SD, n = 3.

### 3.4 Interaction analysis

**FIGURE 6:**
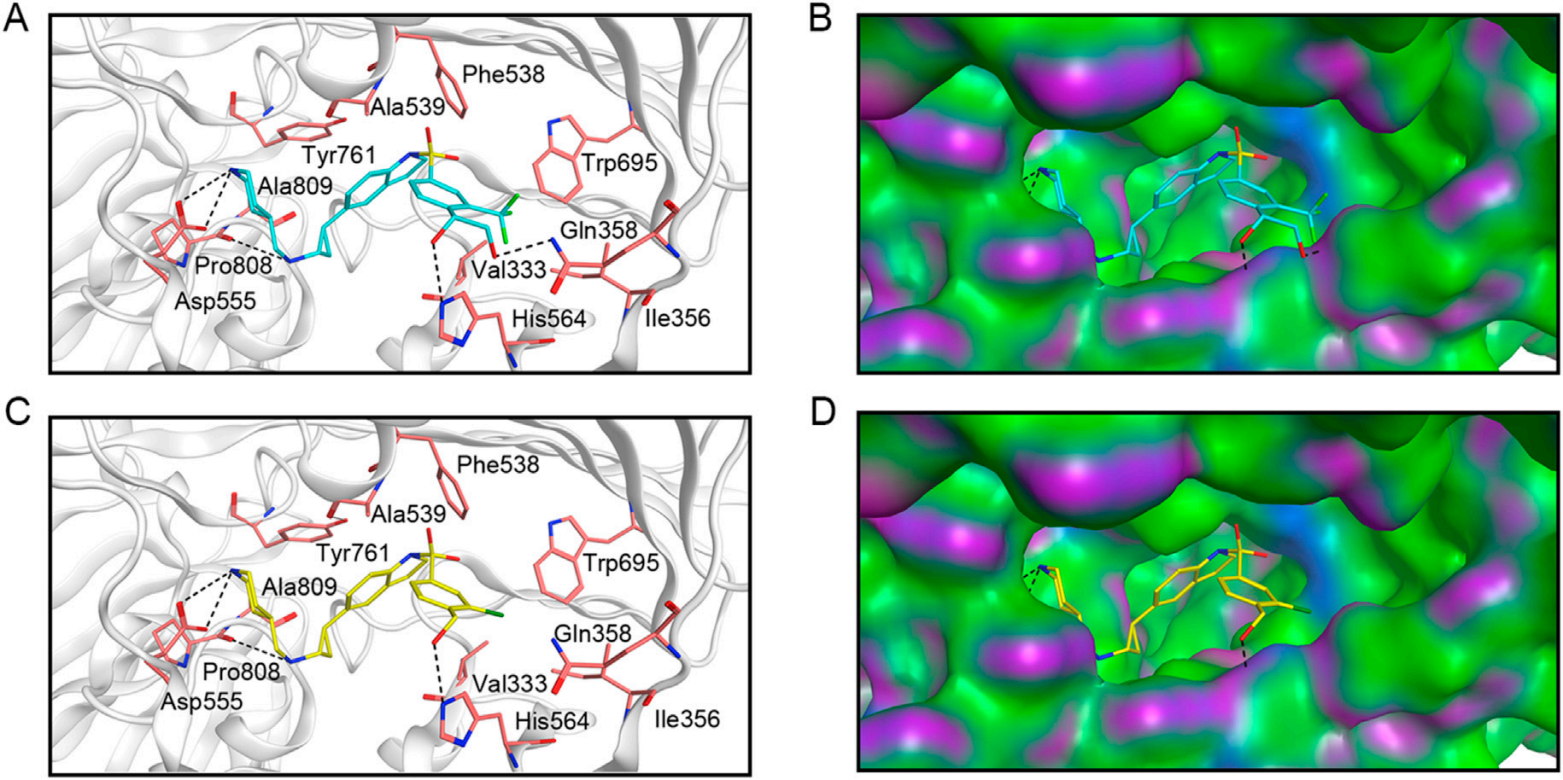
The predicted docking poses and binding surface of LTM-1 and LTM-3 in the active site of LSD1, respectively. **(A)** and **(B)** represent LTM-1; **(C, D)** represent LTM-3. The structure of LTM-1 is represented by cyan sticks. The structure of LTM-3 is depicted by yellow sticks. Key amino acids in the active site are represented by pink sticks and annotated with three-letter amino acid codes. The surface of the LSD1 protein is rendered with hydrogen bond regions (purple), hydrophobic regions (green), and moderately polar regions (blue).

Given that LTM-1 and LTM-3 were identified as having the most favorable docking scores for binding to LSD1 and exhibited the highest potency in *in vitro* enzyme inhibition assays among the six candidates evaluated. Consequently, we conducted an analysis to elucidate the potential binding modes of LTM-1 and LTM-3 with LSD1. As illustrated in Figures 6A, C, LTM-1 and LTM-3 engaged in hydrogen bonding with the critical amino acid residues Asp555, Pro808, and His564 of LSD1. Additionally, they both formed hydrophobic interactions with Val333, Phe538, Ala539, Tyr761, and Trp695 of LSD1. It is noteworthy that LTM-1 established an additional hydrogen bond with Gln358, potentially contributing to its superior binding affinity and inhibitory efficacy. Additionally, the surface topography of the LSD1’s active site, as depicted in Figures 6B, D, revealed a pronounced geometric congruence with the molecular contours of LTM-1 and LTM-3, indicative of an optimal shape complementarity within the binding pocket.

### 3.5 MD simulation

**FIGURE 7:**
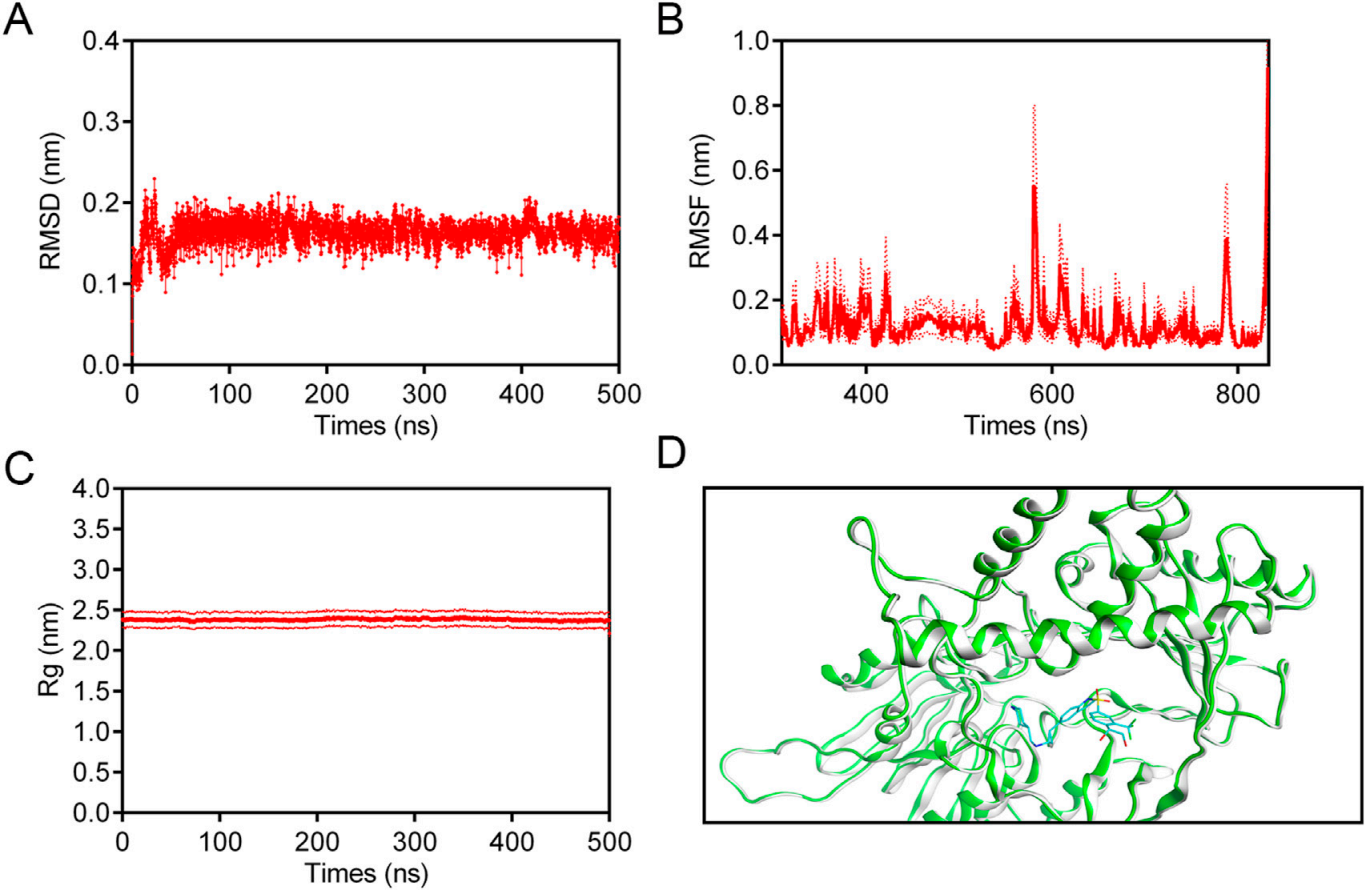
MD simulation of LSD1 in complex with LTM-1. **(A)** RMSD of LSD1-LTM-1 complex. **(B)** RMSF of LSD1 residues in LSD1-LTM-1 complex. **(C)** Rg of LSD1 in the complex of LSD1-LTM-1. **(D)** The conformational changes induced upon binding of the LTM-1 (represented in cyan) to the LSD1 protein (represented in green and gray). The data are presented as the mean ± SD, n = 3. Error bars representing the standard deviation are indicated by red dashed lines.

To determine the binding affinity and persistence of LTM-1 with LSD1, comprehensive molecular dynamics (MD) simulations were conducted over a 500 ns timescale using GROMACS software (version 2021.5). Trajectory analyses indicated that for the LSD1-LTM-1 complex, the root mean square deviation (RMSD) displayed a moderate initial rise, subsequently stabilizing within the range of 0.14 nm–0.20 nm after approximately 7 ns (Figure 7A). This observation suggests that LTM-1 exhibits stable binding with LSD1. Additionally, Figure 7B presents the root mean square fluctuation (RMSF) of LSD1 residues over the 500 ns simulation period. The RMSF values for key residues, including Val333, Phe538, Ala539, Asp555, His564, Trp695, Tyr761, and Pro808, were all below 0.15 nm, which play a crucial role in binding, exhibit minimal fluctuation and maintain stable binding. Furthermore, the radius of gyration (Rg) values for the LSD1-LTM-1 complex exhibited fluctuations of less than 0.1 nm, suggesting that the protein maintained its structural compactness throughout the simulation (Figure 7C). Based on the obtained structural analysis and RMSD values, the conformational stability of the LSD1-LTM1 complex was also analyzed. As shown in Figure 7D, compared with the initial pose in the LSD1-LTM-1 complex, the superposition of the initial pose and the binding-induced pose did not show any conformational changes, and the RMSD value of the LSD1-LTM-1 complex before and after dynamic simulation was less than 0.5 Å, indicating that the LSD1-LTM-1 complex is in a stable conformation. Collectively, these findings substantiate the stable binding of LTM-1 to the LSD1 active site throughout the entire simulation timeframe.

### 3.6 Binding free energy calculation using MM/PBSA method

In this study, the MM/GBSA method was employed to evaluate the strength and stability of interactions between the LSD-1 protein and the LTM-1, performing free energy calculations. Detailed energy components are provided in Table 2. The results showed that the LSD-1-LTM-1 complex had a ΔG_total_ energy of −8,995.02 ± 210.65 kcal/mol, and the electrostatic energy of the LSD-1-LTM-1 complex was −29,188.78 ± 146.72 kcal/mol. The lower the electrostatic energy of the LSD-1-LTM-1 complex, the stronger its binding stability. Further analysis revealed that the electrostatic energy plays a crucial role in providing maximum stability during the binding process, as its contribution to the total binding free energy is significantly greater than the other components. This confirms its importance in the affinity of LTM-1 with the target protein LSD-1.

**TABLE 2:** MM/GBSA energy components of the LSD-1-LTM-1 complex expressed in kcal/mol.

Energy component	LSD-1-LTM-1
△G_total_	−8,995.02 ± 210.65
Van der Waals	−3,327.19 ± 25.94
Electrostatic	−29188.78 ± 146.72
Polar solvation	−4,621.30 ± 126.66
Non-polar solvation	156.05 ± 2.35
Net gas phase	−4,529.77 ± 168.30
Net solvation	−4,465.26 ± 126.69

### 3.7 *In vitro* cancer cell assays

To further evaluate the *in vitro* anti-proliferative effects of LTMs 1–6, we conducted an experiment utilizing the MTS assay to assess the cytotoxicity of these compounds against LSD1-addicted MV-4-11 leukemia cells and LSD1-non-sensitive RPMI-8226 cells. The results, as presented in Table 3, indicated that LTMs 1–6 demonstrated significant anti-proliferative activity against the LSD1-addicted MV-4-11 cells compared to GSK2879552 (IC_50_ = 1.17 ± 0.28 μM), with LTM-1 showing particularly potent effects (IC_50_ = 0.16 ± 0.01 μM). It was noteworthy that the impact of LTMs 1–6 on the LSD1-non-sensitive RPMI-8226 cells was markedly weaker (IC_50_ > 100 μM). Taken together, the potent inhibition of LSD1 by LTM-1 shows its potential as a therapeutic candidate for AML. Additionally, we assessed the antiproliferative activity of LTM-1 against other AML cell lines, including MOLT-4, MOLM-16, HAL-01, and HL-60, to confirm its broader applicability. As shown in Supplementary Table S2, LTM-1 demonstrated significant antiproliferative effects across these AML cell lines (IC_50_ = 0.19–0.39 μM). These findings indicate that LTM-1 not only exerts potent antiproliferative activity against the MV-4-11 cell line but also shows a broad spectrum of anticancer activity against other AML cell lines.

**TABLE 3:** Antiproliferative activities of LTMs 1–6 against human MV-4-11 and RPMI-8226 cells by MTS assay.

Compounds	IC_50_ (μM)	
	MV-4-11	RPMI-8226
LTM-1	0.16 ± 0.01	>100
LTM-2	0.74 ± 0.06	>100
LTM-3	0.32 ± 0.04	>100
LTM-4	0.63 ± 0.05	>100
LTM-5	0.51 ± 0.03	>100
LTM-6	0.98 ± 0.07	>100
GSK2879552	1.17 ± 0.28	21.26 ± 2.69

### 3.8 Antitumor activity of LTM-1 *in vivo*

**FIGURE 8:**
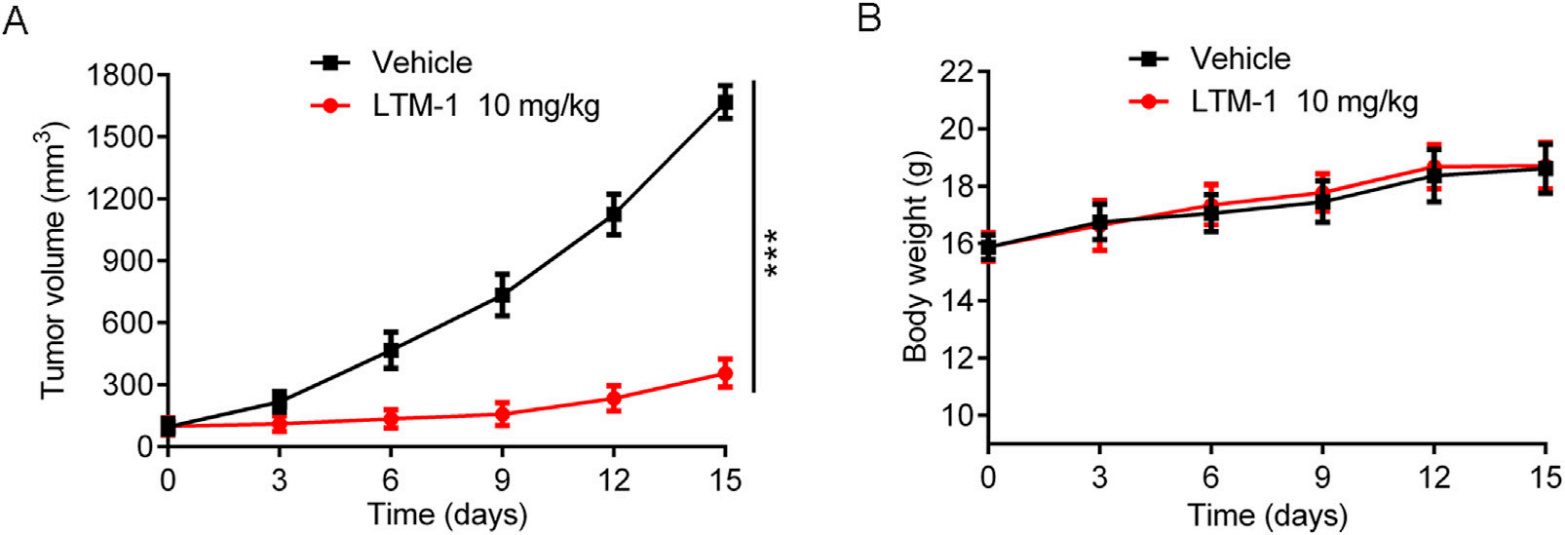
Evaluation of *in vivo* anti-tumor activity of LTM-1. **(A)** Changes in tumor volume. **(B)** Body weight of mice. Results are expressed as means ± SD (n = 3). ***p < 0.001, as compared to the vehicle group.

Considering the outstanding inhibitory effect of LTM-1 on LSD1 protein and MV-4-11 cell proliferation, the MV-4-11 xenograft model was used to further evaluate the *in vivo* anti-tumor activity of LTM-1. Tumor-bearing mice were randomly treated with the vehicle group and 10 mg/kg of LTM-1. As depicted in Figure 8A; Supplementary Figure S1, the tumor growth in mice was significantly reduced upon treatment with LTM-1, compared to the control group. Furthermore, throughout the experimental process, the nude mice in two groups were slowly gaining weight, with no significant differences (Figure 8B), suggesting that treatment with LTM-1 did not induce severe systemic side effects.

We conducted an additional assessment of the potential toxicity of LTM-1. The levels of alanine aminotransferase (ALT), aspartate aminotransferase (AST), blood urea nitrogen (BUN), and creatinine (CRE) in mouse serum were measured to evaluate the functional status of the liver and kidneys, respectively. As illustrated in Supplementary Figure S2, compared to the vehicle control group, there were no significant changes in the levels of ALT, AST, BUN and CRE among mice treated with various doses of LTM-1. This indicates that LTM-1 does not exert notable toxic effects on the liver or kidneys in mice. In summary, while effectively suppressing tumor growth, LTM-1 demonstrates no apparent toxic impact on major organs such as the liver and kidneys in mice. These findings suggest that LTM-1 possesses a favorable safety profile and potential clinical application value.

## 4 Discussion

Acute myeloid leukemia (AML) is a formidable heterogeneous malignant hematologic neoplasm. An increasing body of research has confirmed that lysine-specific demethylase 1 (LSD1) is a key regulatory factor in the self-renewal of leukemic stem cells. Although several LSD1 inhibitors have been reported, they have exhibited unsatisfactory targeting specificity and concerning side effects. Consequently, there is an urgent clinical demand for the development of highly selective and potent LSD1 inhibitors. In this study, an integrated strategy combining pharmacophore modeling, docking screening, MD simulation, and biological validation was employed to identify six LSD1 inhibitor (LTMs 1–6). The results of *in vitro* enzymatic inhibition assays revealed that all six compounds demonstrated potent nanomolar-level inhibitory activity against LSD1. Moreover, the weak inhibitory capacity of LTMs 1–6 against LSD2 underscored their excellent target selectivity. Particularly, LTM-1 and LTM-3 exhibited the most pronounced inhibitory effects on LSD1 and the highest selectivity for LSD1 over LSD2. Then, subsequent interaction analyses indicated that both LTM-1 and LTM-3 formed hydrogen bond interactions with key residues within the LSD1 active site, including Asp555, Pro808, and His564, and also engaged in hydrophobic interactions with Val333, Phe538, Ala539, Tyr761, and Trp695. Additionally, LTM-1 maintained a stable binding state with LSD1 throughout the entire 500 ns MD simulation process. Furthermore, the results of the cell proliferation activity assessment demonstrated that LTMs 1–6 possess strong cytotoxicity against the LSD1-addicted MV-4-11 cells, while having negligible effects on the LSD1-insensitive RPMI-8226 cells, with LTM-1 exhibiting the most potent ability to inhibit cancer cell proliferation. Notably, in the MV-4-11 xenograft model, LTM-1 displayed excellent *in vivo* antitumor activity without significant toxic side effect. In comparison to other LSD1 inhibitors in clinical trials, such as SP-2577 and GSK2879552, LTM-1 has shown superior enzyme inhibition and selectivity against LSD1 (Liu et al., 2023). This comprehensive comparison underscores the potential of LTM-1 as a promising lead compound for preclinical research of AML and reinforces the significance of our research in the context of ongoing clinical trials. While LTM-1 shows promising results in inhibiting AML progression, we acknowledge the need for further structural optimization to improve its pharmacokinetics and minimize toxicity. Therefore, we propose a detailed plan for future structural modifications, which involves substituting the trifluoromethyl group in LTM-1 with other halogens like chlorine, bromine, or iodine. This modification aims to alter the compound’s lipophilicity, potentially enhancing its pharmacokinetic properties and reducing toxicity, thereby providing valuable insights for designing more effective and safer LSD1 inhibitors. Additionally, the virtual screening methodologies utilized in this study, comprising pharmacophore modeling, docking screening, and molecular dynamics (MD) simulations, are conducive to the expeditious identification of novel therapeutics. This methodological framework can be adapted to recognize potential inhibitors from other natural databases and FDA marketed drug databases, so that discovering other novel and efficient natural active small molecules and marketed drugs targeting LSD1. Consequently, it constitutes a potent instrument in the nascent stages of drug discovery, facilitating the identification of target compounds and hastening the lead optimization process.

## 5 Conclusion

In summary, we have designed and identified a series of new potent LSD1 inhibitors (LTMs 1–6). In particular, LTM-1 exhibited the most prominent nanomolar enzyme inhibition ability and strongest inhibition selectivity against LSD1. Our *in vitro* and *in vivo* assays have shown that LTM-1 significantly inhibits the progression of AML without notable toxic side effects, positioning it as a highly promising lead compound for preclinical AML research. While these findings are promising, we recognize the necessity for further experimental validation to substantiate the safety and efficacy of LTM-1 before advancing to preclinical development. As we continue to optimize the structure of LTM-1 in our laboratory, we are also looking ahead to further enhance its potency and selectivity through additional structural modifications. We plan to conduct extensive pharmacokinetic studies to fully characterize LTM-1, ensuring its safety and efficacy. Moreover, we are committed to moving our preclinical findings towards clinical applications and will explore the potential of LTM-1 and its analogs as dual-target inhibitors to improve therapeutic outcomes. We believe that the integration of computational modeling and experimental validation, as demonstrated in this study, will streamline the drug discovery process and significantly contribute to the development of new treatments for AML. The structure-based virtual screening, molecular dynamics simulation, and biological evaluation methods outlined here offer a novel strategy for designing and optimizing more effective and selective LSD1 inhibitors, setting the stage for future research directions that will build upon our current findings.

## Data Availability

The original contributions presented in the study are included in the article/Supplementary Material, further inquiries can be directed to the corresponding authors.
