# Supplementary material for: Discovery of highly potent and novel LSD1 inhibitors for the treatment of acute myeloid leukemia: structure-based virtual screening, molecular dynamics simulation, and biological evaluation
Source: Front Pharmacol. 2025 Feb 27;16:1510319. doi: 10.3389/fphar.2025.1510319 (PMC11903733; doi:10.3389/fphar.2025.1510319)
Supplement: Supplementary file 1 [file DataSheet1.docx]

**Supplementary Material**

**Discovery of highly potent and novel LSD inhibitors for the treatment of acute myeloid leukemia: structure-based virtual screening, molecular dynamics simulation, and biological evaluation**

1. **Supplementary Figures and Tables**

**1.1 Supplementary figures**

**
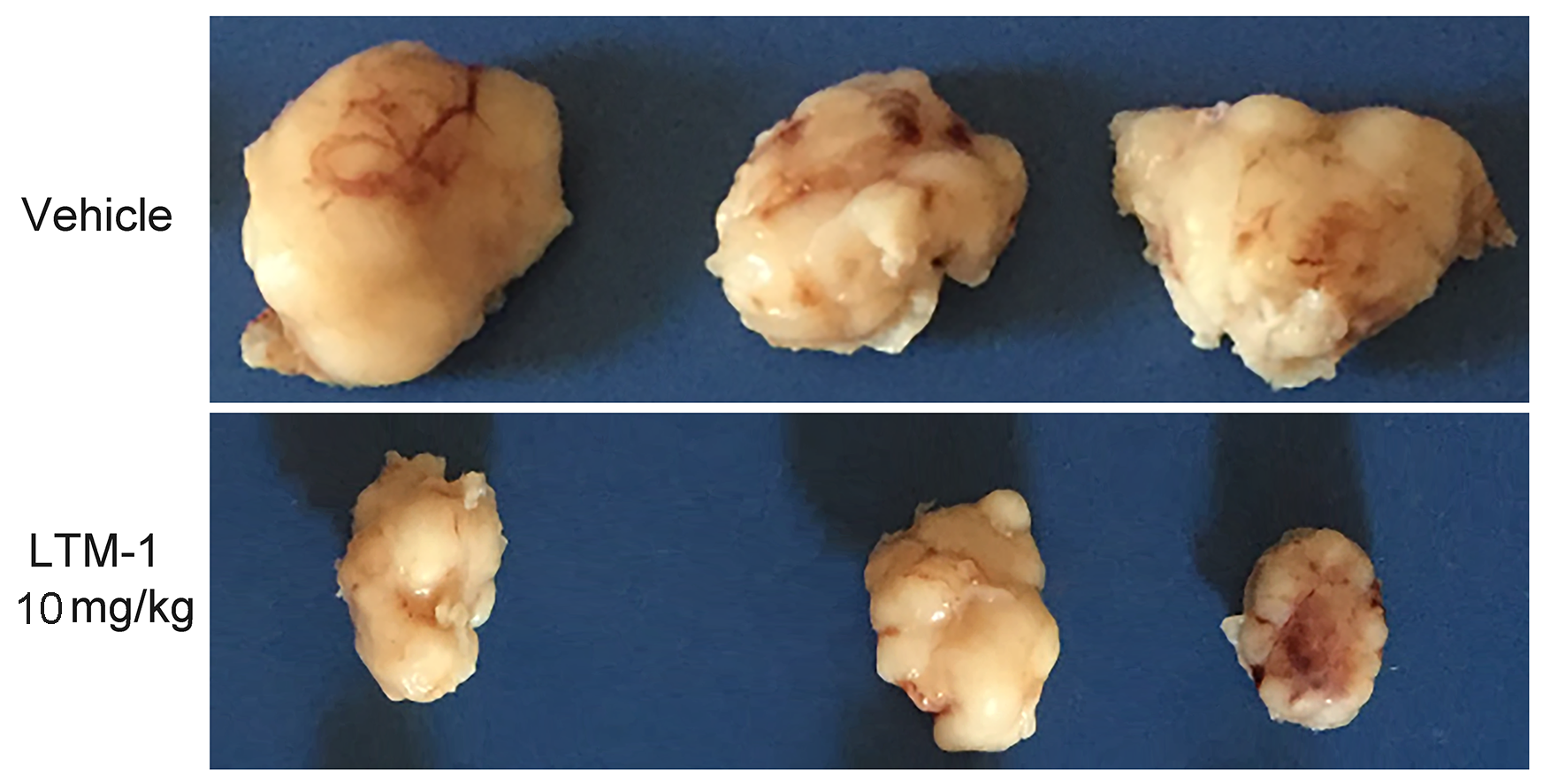
**

**Supplementary Figure S1.** Image of tumors on the 15th day of treatment with vehicle group and 10 mg/kg of LTM-1.


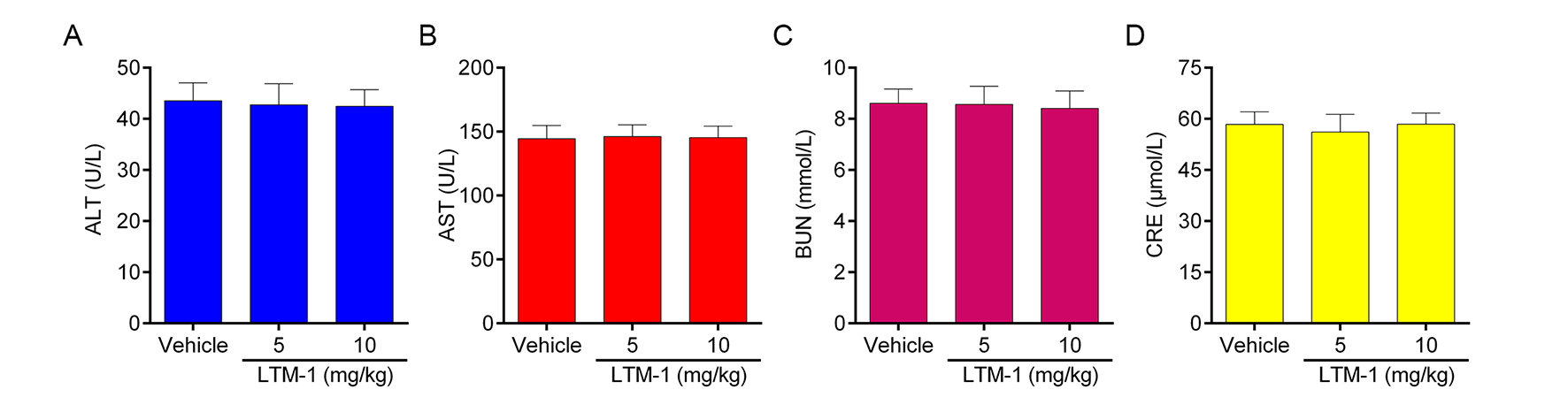


**Supplementary Figure S2.** LTM-1 shows no significant adverse effects in mice. (A) Alanine aminotransferase (ALT) levels were measured in the serum for each group of mice. (B) Aspartate aminotransferase (AST) levels were measured in the serum for each group of mice. (C) Blood urea nitrogen (BUN) levels were measured in the serum for each group of mice. (D) Creatinine (CRE) levels were measured in the serum for each group of mice. Data are presented as mean ± SD, n = 6.

**1.2 Supplementary tables**

**Supplementary Table S1.** Selectivity testing of LTM-1 on a panel of kinases.

| **Target** | **IC_50_ (μM)** | **Target** | **IC_50_ (****μM)** | **Target** | **IC_50_ (μM)** |
| --- | --- | --- | --- | --- | --- |
| ABL1 | > 10 | FES | > 10 | LTK | > 10 |
| ABL2 | > 10 | FGFR1 | > 10 | LYN | > 10 |
| AXL | > 10 | FGFR2 | > 10 | MERTK | > 10 |
| BLK | > 10 | FGFR3 | > 10 | MET | > 10 |
| BMX | > 10 | FGFR4 | > 10 | MST1R | > 10 |
| BTK | > 10 | FGR | > 10 | MUSK | > 10 |
| CSF1R | > 10 | FRK | > 10 | NTRK1 | > 10 |
| PLK1 | > 10 | FYN | > 10 | NTRK2 | > 10 |
| DDR1 | > 10 | PIM1 | > 10 | NTRK3 | > 10 |
| DDR2 | > 10 | RAF1 | > 10 | PDGFRA | > 10 |
| ALK | > 10 | ROS1 | > 10 | PDGFRB | > 10 |
| EPHA1 | > 10 | ZAK | > 10 | PTK2 | > 10 |
| EPHB1 | > 10 | ITK | > 10 | CDK1 | > 10 |
| EPHB2 | > 10 | JAK1 | > 10 | CDK2 | > 10 |
| EPHB3 | > 10 | JAK2 | > 10 | CDK3 | > 10 |
| EPHB4 | > 10 | JAK3 | > 10 | CDK4 | > 10 |
| ERBB2 | > 10 | KDR | > 10 | CDK5 | > 10 |
| ERBB4 | > 10 | KIT | > 10 | CDK6 | > 10 |
| CDK8 | > 10 | CDK13 | > 10 | CDK7 | > 10 |
| CDK12 | > 10 | CDK19 | > 10 | PLK2 | > 10 |

**Supplementary Table S2.** Antiproliferative activity of LTM-1 against other AML cells assessed as growth inhibition measured after 72 h.

| **Name** | **IC_50_ (μM) ^a^** | | | |
| --- | --- | --- | --- | --- |
|  | **MOLT-4** | **MOLM-16** | **HAL-01** | **HL-60** |
| LTM-1 | 0.19±0.01 | 0.27±0.02 | 0.25±0.01 | 0.39±0.03 |

^a^IC_50_ (μM) is the concentration of compound needed to reduce cell growth by 50% after 72 h treatment with LTM-1.
